# Completeness of Electronic Dental Records in a Student Clinic: Retrospective Analysis

**DOI:** 10.2196/13008

**Published:** 2019-03-21

**Authors:** Seth Aaron Levitin, John T Grbic, Joseph Finkelstein

**Affiliations:** 1 Division of Foundational Sciences Columbia University College of Dental Medicine New York, NY United States; 2 Department of Population Health Science and Policy Icahn School of Medicine at Mount Sinai New York, NY United States

**Keywords:** electronic medical records, patient record completeness, dentistry

## Abstract

**Background:**

A well-designed, adequately documented, and properly maintained patient record is an important tool for quality assurance and care continuity. Good clinical documentation skills are supposed to be a fundamental part of dental student training.

**Objective:**

The goal of this study was to assess the completeness of electronic patient records in a student clinic.

**Methods:**

Completeness of patient records was assessed using comparative review of validated cases of alveolar osteitis treated between August 2011 and May 2017 in a student clinic at Columbia University College of Dental Medicine, New York, USA. Based on a literature review, population-based prevalence of nine most frequently mentioned symptoms, signs, and treatment procedures of alveolar osteitis was identified. Completeness of alveolar osteitis records was assessed by comparison of population-based prevalence and frequency of corresponding items in the student documentation. To obtain all alveolar osteitis cases, we ran a query on the electronic dental record, which included all cases with diagnostic code Z1820 or any variation of the phrases “dry socket” and “alveolar osteitis” in the notes. The resulting records were manually reviewed to definitively confirm alveolar osteitis and to extract all index items.

**Results:**

Overall, 296 definitive cases of alveolar osteitis were identified. Only 22% (64/296) of cases contained a diagnostic code. Comparison of the frequency of the nine index categories in the validated alveolar osteitis cases between the student clinic and the population showed the following results: severe pain: 94% (279/296) vs 100% (430/430); bare bone/missing blood clot: 27% (80/296) vs 74% (35/47) to 100% (329/329); malodor: 7% (22/296) vs 33%-50% (18/54); radiating pain to the ear: 8% (24/296) vs 56% (30/54); lymphadenopathy: 1% (3/296) vs 9% (5/54); inflammation: 14% (42/296) vs 50% (27/54); debris: 12% (36/296) vs 87% (47/54); alveolar osteitis site noted: 96% (283/296) vs 100% (430/430; accepted documentation requirement); and anesthesia during debridement: 77% (20/24) vs 100% (430/430; standard of anesthetization prior to debridement).

**Conclusions:**

There was a significant discrepancy between the index category frequency in alveolar osteitis cases documented by dental students and in the population (reported in peer-reviewed literature). More attention to clinical documentation skills is warranted in dental student training.

## Introduction

A clinical record is a fundamental part of patient care delivery [1]. Its completeness is important for many reasons. A record’s main purpose is to serve as a means of communication among providers themselves and between providers and their colleagues [2]. Clinical decision support is dependent on accurate and complete dental records [3], which also aid in the evaluation of a patient’s care [4]. In the event of a lawsuit, the record serves as evidence [5] and its contents are necessary to determine whether the diagnosis and treatment met appropriate standards [6]. With the expansion of dental informatics applications, it is even more essential to have a complete record in order to ensure proper analysis and results in outcomes research [5]. However, there are known issues with the completeness of electronic medical records (EMRs).

In the past decade, electronic patient records became a ubiquitous part of dental care delivery [7]. Oral health data accumulated in the process of clinical care represent a rich and readily available recourse for scientific investigation and data analytics [8]. Recent analysis of electronic dental records (EDRs) helped identify predictors of implant survival [9] as well as the prevalence and risk factors of peri-implantitis [10]. Application of machine learning techniques [11] and temporal analytics [12] resulted in new opportunities for knowledge discovery and predictive analytics [13]. With the increasing use of EMRs as an important resource for scientific discovery, potential barriers for secondary analysis of EMR have been recognized [14]. Completeness of EMR is one of the most frequently discussed issues that may limit the use of EMR data for clinical and population health research [15]. This issue was reported to be particularly relevant in the evaluation of medical student documentation [16]; however, no systematic assessment of the completeness of EMR in dental student clinics has been performed.

Since the widespread introduction of EMRs, numerous studies have indicated gaps in documentation. A study at the University of Michigan examined whether there were differences in the reported eye symptoms between EMRs and eye symptom questionnaires that patients fill out. Exact agreement was found in only 23.5% (38/162) of cases. In cases where patients reported three or more symptoms, data from the eye symptom questionnaire always varied from the EMR data [17]. A similar study at the Mayo Clinic compared symptoms of chest pain, dyspnea, and cough between information forms patients received prior to the appointment and EMRs, for the purpose of identifying stable angina pectoris. They found that the two documents had varying levels of positive agreement (ratio): 74 for chest pain, 70 for dyspnea, and 63 for cough [18]. Researchers at Duke University analyzed records for completeness for quality purposes, but concluded that improper documentation for colorectal cancer impeded their ability to accurately calculate patient performance. Of the 499 patients eligible for the analysis, only 66 had sufficient documentation. In addition, only 86% (427/499) of EMRs confirmed a diagnosis, 29% (143/499) were missing the age, and only 38% (188/499) stated the TMN stage [19].

Other studies indicated that the quality of documents in EDRs may be suboptimal [5,20]. However, there is a lack of systematic studies on the completeness of EDRs. The goal of this project was to review the documentation quality of EDRs in a dental school clinic.

## Methods

### Data Source

EDRs of patients examined at Columbia University’s College of Dental Medicine were analyzed.

### Data Collection

The study was a retrospective analysis of EDRs from patients with dry sockets diagnosed at Columbia University’s College of Dental Medicine between August 2011 and May 2017. We ran a query on the college’s database to find EDRs containing diagnostic code Z1820, the phrases “dry socket” and “alveolar osteitis,” or a variation of those two phrases. All queries were performed using structured query language in the Oracle database containing data from an EDR system called axiUm (Exan Group, Las Vegas, NV).

For the purpose of this study, a dry socket was defined by a diagnostic Z code at the initial encounter. Alternatively, it was defined by the presence of a key word in a note combined with clinical evidence and explicit documentation of the patient with alveolar osteitis or dry socket. Z codes are represented by a list of diagnostic codes and terms developed for use with EDR, as previously described [21].

After reviewing the literature on dry socket [22-28], we compiled a list of 17 criteria related to dry socket. *Five* were related to treatment: curettage, irrigation, anesthesia, intra-alveolar medication, and medication. *Three* were symptoms: pain, radiating pain, and tenderness on palpation. *Nine* were signs: lack of blood clot, malodor, low-grade fever, bare bone, lymphadenopathy, pus, erythema, inflamed gingiva/socket, and debris. The *last* category was the socket site of dry socket. Severe pain was a necessary symptom for dry socket diagnosis [25,28].

### Data Analysis

A dental student reviewed each of the query results to confirm the presence of a dry socket. The cases were also analyzed for the presence of any of the 17 criteria mentioned above. Pertinent positive and negative results for dry socket criteria were both recorded for further analysis. The student then reviewed the available literature on dry socket statistics to determine the prevalence of symptoms in the literature. Both the positive and negative criteria were compared to the baseline figures from this literature.

## Results

**Figure 1:**
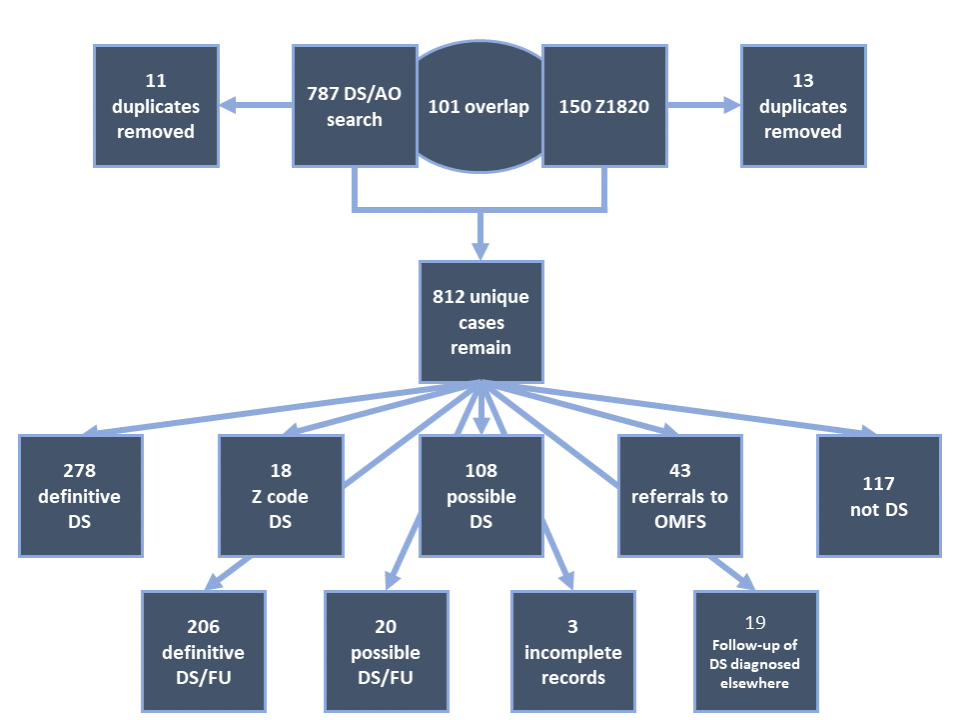
Breakdown of the reviewed cases. DS: dry socket; AO: alveolar osteitis; FU: follow-up; OMFS: Department of Oral and Maxillofacial Surgery.

A total of 150 records with diagnostic Z codes were identified, and another 787 records were identified by searching for a mention of dry socket or alveolar osteitis in the notes (Figure 1). Both queries resulted in a number of duplicate cases, which were removed. The dry socket/alveolar osteitis dataset had 11 duplicate cases, and the Z code data set contained 13 duplicate cases. In addition, 101 of the remaining cases overlapped (ie, had both diagnostic Z codes *and* dry socket or alveolar osteitis in the notes). These overlapping cases were not counted twice in the study. After removing duplicates and overlapping cases, the final number of combined cases was 812.

Cases were then categorized based on the dental record content (Table 1). An explicit diagnosis of dry socket was found in 278 EMRs. Another 18 EMRs had a diagnostic Z code and the patient received treatment for dry sockets, but the dentist never explicitly stated that the patient presented with a dry socket; these were also included as definitive dry sockets for the purpose of this study. Totally, 296 cases (of 812, 36.5%) were categorized as definitive dry sockets, and 108 cases (of 812, 13.3%) of possible dry sockets had notes of treatment of dry sockets, but the dentist did not include a diagnostic code or any definitive diagnoses of dry socket in the note. This misdocumentation is important, but could not be included in the data, as there is no way to confirm the patient had a dry socket from the records. The remaining cases were classified as follows:

- Follow-up treatment of both definitive dry sockets and possible dry sockets (226/812, 27.8%).
- Cases that were definitively classified as nondry sockets: These patients’ records were included in the query because they contained the key word to rule out the diagnosis, history of previous dry socket, mention of dry socket paste, or similar reference (117/812, 14.4%).
- Referrals to the oral surgery clinic: Such referrals for further evaluation were also a common finding. The referrals were not followed up to determine if these patients were later seen in Columbia (43/812, 5.3%). An additional segment of charts belonged to patients whose only encounter populated in our search was for a follow-up in our clinic for a dry socket (19/812, 2.3%). The final and smallest group of charts contained a Z code, but no note was found in the system for the encounter. This may have been a kink in the system or oversight by the provider (3/812, 0.4%).

Definitive dry socket cases were analyzed for correctness of EMR documentation. The demographics of patients of the 296 records are outlined in Table 2. Women (196/296, 66.3%) were affected by dry socket more frequently than men. The age group with the highest frequency of dry socket included adults between the ages of 20 and 39 years (146/296, 49.3%). Table 3 demonstrates the extraction characteristics of the definitive dry sockets studied. Molars were the most frequent sites for dry socket, with the third molar represented in 37.5% of the cases (111/296). Tooth extraction was performed under the supervision of faculty from the Department of Oral and Maxillofacial Surgery in 82.8% (245/296) cases. About half of the tooth extraction procedures were carried out by predoctoral students (145/296, 46.3%) and the remaining extractions were performed by postdoctoral students, the majority of whom were represented by residents of the Department of Oral and Maxillofacial Surgery. A similar pattern was observed for location and provider type for the tooth extraction follow-up visits during which dry socket was diagnosed and treated.

**Table 1:** Categories of classifications.

Category	Description	n
Definitive dry socket	Diagnostic code with signs, symptoms, and treatment, or explicit clinical diagnosis	296
Dry socket follow-up	Follow-up to case classified as definitive dry socket, possible dry socket, or other treatment	230
Possible dry socket	Presented with symptoms and treated for dry socket, but no explicit diagnosis or Z code	108
Referral	Referred to the Department of Oral and Maxillofacial Surgery	43
Not dry socket	Diagnosis excluded by provider, history of previous dry socket in notes, or similar finding	117
Dry socket treatment follow-up or missing other documentation	Only the follow-up on previous treatment was contained in the query	15
Incomplete notes	Notes were not populated but contained the Z code	3

**Table 2:** Patient demographics.

Characteristic		n (%)	
**Gender**			
	Male		100 (33.7)
	Female		196 (66.3)
**Age (years)**			
	<20		11 (3.7)
	20-39		146 (49.3)
	40-59		82 (27.7)
	60-79		53 (17.9)
	>80		4 (1.4)
**Ethnicity**			
	African American		23 (7.8)
	Asian		4 (1.4)
	Caucasian		9 (3.0)
	Hispanic		120 (40.5)
	Other		139 (47.0)
	Not disclosed		1 (0.3)
**Language**			
	English		195 (65.88)
	Spanish		68 (22.97)
	Other		30 (10.14)
	Arabic		2 (0.68)
	Russian		1 (0.34)

Two general types of misdocumentation were encountered and calculated while analyzing the 296 cases of definitive dry socket (Table 4). The first was any missing gross documentation necessary for diagnosis of dry socket to support the clinical diagnosis of this condition and to provide documentation necessary for patient follow-up and care continuity. These included documented pain, visible bone, or lack of blood clot. We also included the missing socket position when diagnosing a dry socket. Of the 296 cases, 220 (74.3%) were missing at least one of the abovementioned factors. The second type of misdocumentation was the number of cases without a diagnostic Z code. Of the 296 cases, 232 (78.4%) did not contain the diagnostic code. All cases analyzed were documented after the Columbia University College of Dental Medicine implemented diagnostic codes.

**Table 3:** Extraction characteristics.

Characteristics		n (%)
**Extracted tooth**		
	Third molar	111 (37.5)
	First and second molars	125 (42.2)
	Premolars	40 (13.5)
**Department of extraction treatment**		
	Department of Oral and Maxillofacial Surgery	245 (82.8)
	Periodontics	13 (4.4)
	Other	38 (12.8)
**Provider group for extraction**		
	Student	145 (46.3)
	Department of Oral and Maxillofacial Surgery	65 (24.1)
	General practice residency	23 (8.5)
	Advanced education in general dentistry	18 (6.7)
	Periodontics	14 (5.2)
	Other	31 (10.4)
**Location of dry socket treatment**		
	Department of Oral and Maxillofacial Surgery	242 (81.8)
	Advanced education in general dentistry	17 (5.7)
	Periodontics	14 (4.7)
	Other	23 (7.8)
**Provider groups for dry socket diagnosis**		
	Student	143 (48.3)
	Department of Oral and Maxillofacial Surgery	49 (16.55)
	General practice residency	42 (14.19)
	Advanced education in general dentistry	26 (8.78)
	Periodontics	13 (4.39)
	Other	23 (7.8)

Possible misdocumentation was also found in the remaining 516 cases that were not labeled as definitive dry socket. There were 108 cases of patients who presented with symptoms of dry sockets and received treatment for dry sockets. However, their charts did not include a Z code or any definitive diagnoses by the provider. Although many of these patients fulfilled the requirements to be classified with a dry socket, they could not be included in the analysis because they lacked a basic documented confirmation. As previously stated, 206 cases were classified as follow-up appointments to initial treatment of definitive dry socket. From these cases, another category of misdocumentation emerged: 16 of the cases contained a diagnostic Z code, although the actual diagnosis and initial treatment of the dry socket did not contain this code. The latter type of misdocumentation does not have any direct consequences, but reveals a reluctance to use dry socket diagnosis or lack of education regarding diagnostic codes.

The final group of misdocumentation was found in the 19 cases categorized as follow-up to the initial appointment for dry socket, in which the patient’s first documented encounter with the word dry socket or alveolar osteitis was in the follow-up. The clinical note in 14 of these 19 cases indicated that the patient was previously seen in the clinic or emergency room to treat the dry socket. The implication is that these patients were seen and treated for a dry socket previously in the clinic, but the earlier documentation lacked any of the three search terms (dry socket, alveolar osteitis, and Z code) used to identify cases for this analysis.

**Table 4:** Types of misdocumentation encountered (N=296).

Type of misdocumentation	n (%)
Missing documentation necessary for diagnosis (pain, bone visible, or socket position)	220 (74.3)
No Z codes	232 (78.4)
Treated for dry socket and had symptoms, but no definitive diagnosis	108 (36.5)^a^
Dry socket follow-up is Z code but no initial encounter	16 (5.4)^a^
Dry socket follow-up was searchable but not the original case	14 (4.7)^a,b^

^a^These cases were categorized for purposes of misdocumentation but were not included in the analysis of misdocumentation of definitive dry sockets.

^b^Includes 2 from the emergency room.

**Figure 2:**
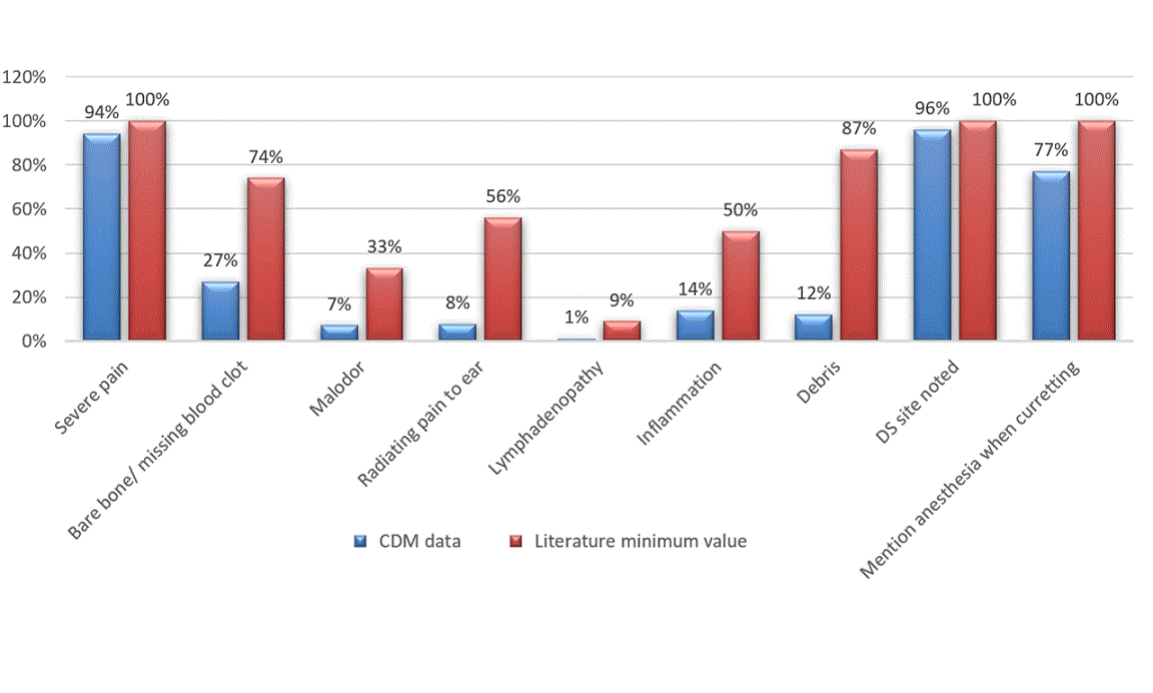
Signs and symptoms in misdocumentation. DS: dry socket; CDM: clinical dental record.

Definitive dry socket signs, symptoms, and treatments completeness compared to the best values available in the literature are presented in Figure 2. Presence of severe pain was considered a necessary symptom for dry socket diagnosis. The clinical notes only contained pain as a symptom in 94% (279/296) of the cases. Bare bone [23], open socket [25], or missing blood clot [26] was mentioned in 74% (35/47) to 100% (329/329) of the dry socket cases. In our clinic, any of these related terms were mentioned in 27% (80/296) of cases. In the 296 definitive dry socket cases, the word “bone” was only found in 50 cases and “clot” was found only 25 times; some of these cases overlapped. These terms are germane and necessary for a diagnosis of dry socket, and the lack of documentation is troublesome. Malodor [22] was documented in 33% (18/54) to >50% of the dry socket cases in the literature [27]; however, its documentation was only present in 7% (22/296) of our cases. Radiating pain toward the ear was present in 56% (30/54) of the cases in the literature [22], but was mentioned only in 7% (24/296) of our cases (documentation of radiating pain alone was included in this number, as we did not require ear or side of face to be documented). Lymphadenopathy [22] was present in 9% (5/54) of the cases in the literature [23], but in only 1% (3/296) of our cases. Inflammation was present in 50% (27/54) of the cases in the literature [22]; however, in the dental record, only 14% (42/296) of cases mentioned inflammation and 15% (44/296) (with some overlap with inflammation cases) mentioned erythema.

A comparison between the notes of predoctoral dental students and postdoctoral faculty and residents is outlined in Table 5. The nine selected signs, symptoms, and other documentation related to dry socket diagnosis were analyzed. Predoctoral students were more likely to properly document the location of the dry socket, while postdoctoral students were more likely to document malodor, presence of debris, and anesthesia administration during socket debridement. Misdocumentation of the remaining categories was equal (both groups were within 1% of one another for the other five categories) as compared to the expected values (Figure 2).

**Table 5:** Comparison between the notes of predoctoral dental students and postdoctoral faculty and residents.

Characteristic	Predoctorate (n=143), n (%)	Postdoctorate (n=151), n (%)
Severe pain	135 (95)	144 (94)
Bare bone/missing blood clot	31 (22)	49 (32)
Malodor	6 (4)	16 (10)
Radiating pain to ear	12 (8)	12 (8)
Lymphadenopathy	1 (1)	2 (1)
Inflammation	21 (15)	21 (14)
Debris	12 (8)	24 (16)
Dry socket site noted	140 (98)	143 (93)
Anesthesia mentioned during debridement	2^a^ (50)	18^b^ (82)

^a^Total for this group is 4 students.

^b^Total for this group is 22 faculty and residents.

## Discussion

Our ultimate finding was that misdocumentation occurs in EDRs to varying degrees within a dental school clinic. The dental records reviewed lack many signs and symptoms that are necessary and expected to be recorded for a diagnosis of dry socket. This misdocumentation was prevalent in the notes of both predoctoral and postdoctoral students. The major limitation of this study, as with other electronic record retrospective studies, was our ability to confirm the diagnosis of dry socket. We also could not confirm whether patients actually had the symptoms omitted. We were forced to rely on the information provided by the documenter.

Evaluation of completeness of symptom documentation in this study followed recent guidance on EMR data quality–assessment methodology [29] that promulgates the use of validated population-based prevalence as a gold standard [30-31]. Following these guidelines, the comparison between the validated symptom prevalence and symptom frequency found in our EDR review was used to identify completeness of symptom documentation in EDR in this study. This approach has been successfully used in a number of previous studies to identify completeness of smoking status records [32], obesity reporting [33], hypertension records [34], and depression prevalence [35]. To minimize bias toward inflating misdocumentation rates, whenever several verified estimates of a population-based frequency for a particular symptom were available, a comparison was made between the lowest population-based frequency and the frequency found in the EDR, as previously described [29-32].

Our findings of the demographics and tooth extraction characteristics among patients with dry socket are congruent with previous reports. As previously determined, women are more likely to have dry socket then men [25,36]. The tooth position is most likely to be a third molar compared to any other individual area [37]. People in their 20s and 30s are at a higher risk for alveolar osteitis [38]. Two major types of misdocumentation that were found and analyzed in this study have been previously mentioned in the literature, including lack of supporting documentation for clinical diagnosis [25,28] and absence of appropriate diagnostic codes [39].

Analysis of characteristics of providers who performed tooth extraction and dry socket diagnosis confirmed the external validity [40] of our study sample, as the resulting characteristics accurately reflected routine dental care delivery patterns for these types of procedures occurring in the student clinic. The majority of the cases were carried out under supervision of a preceptor from the Department of Oral and Maxillofacial Surgery. About half of the extractions and dry socket diagnoses were carried out by predoctoral students, and the rest were performed by postdoctoral residents of whom approximately half were represented by residents of the Department of Oral and Maxillofacial Surgery. The fact that the predoctoral and postdoctoral dental surgeons were equally presented in our analysis supported the unbiased comparison between documentation quality of predoctoral and postdoctoral students.

The results of documentation completeness in EDRs in this study are corroborated by the following reports. In Minnesota, a discrepancy was found between the American Dental Association’s recommendation for dental record accuracy and the actual accuracy in dental practices [41]. In Finland, researchers observed a discrepancy between the quality of treatment a dentist believed he/she provided and the treatment the patient actually received, as contained in the EDR [42]. EMRs have also been shown to have issues with accuracy [43]. In an adult cardiology clinic, researchers discovered “very poor” completeness values for signs such as chest pain and shortness of breath [44]. In a systemic review of EMR completeness in primary care, Thiru [45] found that records of diagnoses with clear clinical criteria had a higher rate of completeness than those without clear criteria. This is relevant to dry socket, a diagnosis with unclear criteria. A trauma center study also found incompleteness of certain categories in the EMR [46]. Similarly, a study conducted with inpatient records at Menelik II Referral Hospital, a government hospital in Addis Ababa, Ethiopia, found “low” EMR completeness compared to the expected standard of 100% [47]. Legal Medical Record Standards stated that “Each Medical Record shall contain sufficient, accurate information to identify the patient, support the diagnosis, justify the treatment, document the course and results, and promote continuity of care among health care providers” [48]. Following this simple instruction can vastly improve the delivery of care.

To resolve the issues with incomplete or inaccurate records, dental education should emphasize more on proper documentation and ensure its incorporation into the clinic routine. Documenters need to remember that their records are not simply for their own convenience, but may serve legal, research-related, or forensic purposes [1,49]. To this end, each record must be complete, without implicit assumptions, and follow a method that makes it easily accessible to any reader. Thierer [50] found an improvement in EDR accuracy by incorporating an in-service intervention for faculty members and a Moodle site course on documentation for students.

Our findings have important implications for future research that uses EMR data. Better understanding of the potential limitations of electronic health record data use promotes fidelity and reproducibility of secondary data analysis [51]. A variety of approaches are being implemented to address the potential limitations of EMR data [52] such as deep learning techniques [53] for imputing missing data, symbolic operations for time interval analytics [54], and calibration to reduce measurement error in prevalence estimates based on EMR data [55]. A growing number of studies employ common data models combined with cross-linked semantic ontologies to harmonize EMR data [56] and confirm with the Findability, Accessibility, Interoperability, and Reusability principles [57].

We believe there are three sequential steps necessary for improving EDRs. The first step is additional training predoctoral and postdoctoral students on the importance of note comprehensiveness. As per a systemic review by the Accreditation Council for Continuing Medical Education, a live intervention with interactive techniques is the most effective way to change a physician’s behavior to influence patient outcomes [58]. Interventions should therefore be constructed accordingly. The next step is adding disease- and condition-specific worksheets to the EDR. The worksheets should contain categories pertinent to the specific diagnosis, with drop-down boxes for the practitioner to complete. This prevents the inadvertent omission of crucial categories. The last step is adding a clinical decision support tool to EDRs. The tool embodies evidence-based dentistry, an approach being adopted by an increasing number of dental schools and practitioners. However, this tool operates properly only if practitioners enter complete and accurate data into EDRs in a way that computers can easily analyze. Thus, strict compliance with the first two steps is critical. This will result in refined EDRs, which can potentially lead to superior and safer delivery of care at a lower cost [59]. Although some information in EDRs may seem largely irrelevant, the EDR is a critical depository of data, with limitless research possibilities. If properly executed, it may improve diagnoses, treatment, and dentistry as a whole.

## Abbreviations

AEGD: advanced education in general dentistry
EDR: electronic dental record
EMR: electronic medical record
GPR: general practice residency
OMFS: Department of Oral and Maxillofacial Surgery
